# Young, healthy males and females present cardiometabolic protection against the detrimental effects of a 7-day high-fat high-calorie diet

**DOI:** 10.1007/s00394-020-02357-3

**Published:** 2020-08-13

**Authors:** Katie L. Whytock, Sam O. Shepherd, Matt Cocks, Anton J. M. Wagenmakers, Juliette A. Strauss

**Affiliations:** Research Institute of Sport and Exercise Science, Liverpool John Moores University, Liverpool, UK

**Keywords:** High-fat, High-calorie, Metabolic health, Sex differences

## Abstract

**Purpose:**

High-fat, high-calorie (HFHC) diets have been used as a model to investigate lipid-induced insulin resistance. Short-term HFHC diets reduce insulin sensitivity in young healthy males, but to date, no study has directly compared males and females to elucidate sex-specific differences in the effects of a HFHC diet on functional metabolic and cardiovascular outcomes.

**Methods:**

Eleven males (24 ± 4 years; BMI 23 ± 2 kg.m^−2^; V̇O_2 peak_ 62.3 ± 8.7 ml.min^−1^.kg^−1^FFM) were matched to 10 females (25 ± 4 years; BMI 23 ± 2 kg.m^−2^; V̇O_2 peak_ 58.2 ± 8.2 ml.min^−1^.kg^−1^FFM). Insulin sensitivity, measured via oral glucose tolerance test, metabolic flexibility, arterial stiffness, body composition and blood lipids and liver enzymes were measured before and after 7 days of a high-fat (65% energy) high-calorie (+ 50% kcal) diet.

**Results:**

The HFHC diet did not change measures of insulin sensitivity, metabolic flexibility or arterial stiffness in either sex. There was a trend towards increased total body fat mass (kg) after the HFHC diet (+ 1.8% and + 2.3% for males and females, respectively; *P* = 0.056). In contrast to females, males had a significant increase in trunk to leg fat mass ratio (+ 5.1%; *P* = 0.005).

**Conclusion:**

Lean, healthy young males and females appear to be protected from the negative cardio-metabolic effects of a 7-day HFHC diet. Future research should use a prolonged positive energy balance achieved via increased energy intake and reduced energy expenditure to exacerbate negative metabolic and cardiovascular functional outcomes to determine whether sex-specific differences exist under more metabolically challenging conditions.

## Introduction

A western lifestyle is characterised by regular consumption of readily available energy-dense foods [1], often paired with sedentary behaviour throughout the day [2] and/or minimal amounts of exercise [3]. These behavioural patterns are associated with an increased risk of obesity, type 2 diabetes (T2D) and cardiovascular disease [4–6]. At present, cardiovascular disease is established as the leading cause of mortality in western countries [7] and T2D vastly increases the risk of cardiovascular complications [8].

Insulin resistance is defined as impaired insulin-stimulated glucose uptake in skeletal muscle and adipose tissue stores often combined with impaired insulin-induced suppression of hepatic glucose production. Both hepatic and skeletal muscle insulin resistance are central to the development of T2D [9] [10]. Metabolic flexibility is closely linked with skeletal muscle insulin sensitivity [11], and is defined as the capacity to switch from high rates of lipid oxidation in the fasted state to an increased rate of carbohydrate oxidation under insulin-stimulated conditions [12]. Metabolic flexibility is often diminished in obese insulin-resistant individuals in comparison to the lean insulin-sensitive state [13].

Whilst obesity itself is commonly associated with insulin resistance and T2D [14], it is now clear that distinct fat patterning is more closely related with disease risk [15]. For example, fat accumulation in the abdominal region, and in particular visceral fat, is strongly related to metabolic disease and systemic inflammation [16–19], whilst peripheral adipose tissue located in the gluteal–femoral regions appears to be protective against metabolic disease [20]. Abdominal adiposity is a typical feature of the male population, with females tending to accumulate fat in the gluteal–femoral region [21]. These differences in adipose tissue storage between sexes likely underpin the sex differences in the risk for developing metabolic disease. Epidemiological studies have shown that the incidence of T2D and cardiovascular disease is lower in females compared to males [7, 22, 23]. Large cross-sectional studies have also shown that males (35–65 years) have a higher prevalence (+ 1.3–1.9%) of elevated fasting glucose in comparison to females [24, 25]. In another cross-sectional study, males (18–32 years) had a reduced blood glucose clearance capacity (− 15%) following an intravenous glucose tolerance test compared to females [26]. Smaller clinical trials investigating insulin sensitivity in BMI and age-matched males and pre-menopausal females (20–45 years) using a hyperinsulinaemic euglycaemic (HE) clamp have established that females exhibit a similar or higher glucose infusion rate per kilogram of body weight (+ 36–47%) when compared to males [27–31], and a markedly higher glucose infusion rate when adjusted per kilogram of lean mass (45–98%) [28, 31].

Despite females being at a reduced risk of developing insulin resistance and cardio-metabolic disease, there is little research investigating the differences in the progression of insulin resistance and negative cardio-metabolic health between the sexes. One approach to investigate this is to provide excess lipid through an acute lipid infusion during a HE clamp, and it has been shown that a 7 h lipid infusion caused less of a reduction in glucose infusion in females (26%) in comparison to males (38%) [32].

Excess lipid exposure from infusion studies may only illustrate acute physiological responses, whereas more prolonged consumption of a high-fat high-calorie (HFHC) diet provides a chronic stimulus to investigate the influence of increased fat and energy intake (creating a positive energy balance) on adipose tissue stores and early abnormalities in metabolic and cardiovascular regulation. In healthy individuals, consumption of a HFHC diet for 5–7 days has been demonstrated to reduce hepatic insulin sensitivity (− 65%) [33] and peripheral insulin sensitivity (− 20%) [34] during a HE clamp. HFHC diets also reduced whole-body insulin sensitivity (− 27%) measured with an oral glucose tolerance test [35] or glucose tolerance (− 11%) measured after ingestion of a mixed meal (112 g carbohydrate in a 771 kcal meal) [36]. Although some of these studies have included female participants [35, 36], to date, no study has directly compared sexes to identify the sex-specific cardiovascular and metabolic responses to a short-term HFHC diet. This is a considerable oversight considering females, compared to males, exhibit higher insulin sensitivity and tend to store fat peripherally, rather than centrally, and therefore appear to be better protected against metabolic disease. Establishing whether females are better equipped to handle lipid overload from a 7-day HFHC diet will be beneficial in underpinning the pathology of T2D in this understudied cohort. Consequently, the aim of this study was to investigate the hypothesis that females matched for age, BMI, cardio-respiratory fitness and habitual activity levels are better protected than males in developing insulin resistance induced by a HFHC diet. We also aim to underpin sex-specific differences in metabolic and cardiovascular functional outcomes (fat mass accumulation, metabolic flexibility, arterial stiffness and blood lipid and liver concentrations) in response to the 7-day HFHC diet.

## Methods

### Subjects and ethical approval

A cohort of young healthy males (*n* = 11) and females (*n* = 11) were matched for BMI (kg.m^−2^), age and VO_2 peak_ [ml.min^−1^.kg (fat-free mass (FFM))^−1^] (see Table 1 for characteristics). VO_2 peak_ was adjusted to FFM to remove the influence of increased fat mass on body mass observed in the female cohort allowing for direct comparison of sexes. The sample size for this experiment was estimated based on previous literature that showed a reduction in glucose tolerance or insulin sensitivity following 7 days of a high-fat (65% energy) high-calorie diet (+ 50% kcal) [35, 36]. Written informed consent was obtained from all participants. The study protocol adhered to the Declaration of Helsinki and was approved by NHS West Midlands, Black Country Research Ethics Committee. All participants were free from cardiovascular or metabolic disorders, physically active (exercising at least 3 times per week for more than 30 min at a time) and non-smokers. Additionally, females were required to have a regular menstrual cycle, not be pregnant or breast-feeding during the study and only using the following contraceptive methods; condoms, diaphragm, IUD (intrauterine device), combined pill. Due to the nature of the dietary intervention, vegetarians and vegans were excluded from participation. During the dietary intervention, one female participant withdrew from participation due to gastro-intestinal discomfort and, therefore, her data were withdrawn from the analyses. Withdrawal of this participant did not affect the group matching of sexes.

**Table 1:** Participants characteristics

	Males	Females
*n*	11	10
Age (years)	24 ± 4	25 ± 4
Body mass (kg)*	72.6 ± 5.3	63.9 ± 9.5
BMI (kg.m^−2^)	23 ± 2	23 ± 2
V̇O_2 peak_ (L.min^−1^)*	3.60 ± 0.59	2.51 ± 0.48
V̇O_2 peak_ (ml.min^−1^.kgFFM^−1^)	62.3 ± 8.7	58.2 ± 8.2
Matsuda ISI	11.6 ± 10.5	9.2 ± 3.4

Data provided as mean ± SD

*BMI* body mass index, *FM* fat mass, *FFM* fat-free mass, *ISI* insulin sensitivity index

*Significant difference between sexes *P* < 0.05

### Pre-experimental procedures

**Fig. 1:**
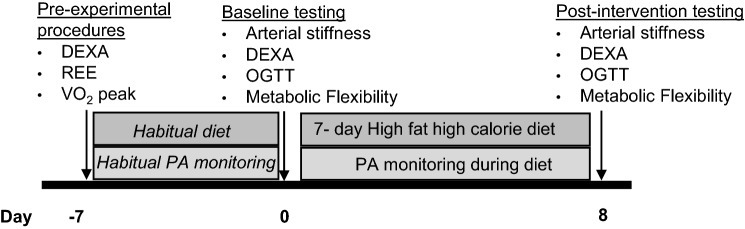
Schematic diagram of study design. *DEXA* dual-energy X-ray absorptiometry, *OGTT* oral glucose tolerance test, *PA* physical activity, *REE* resting energy expenditure

Prior to the start of the study, participants attended the laboratory following an overnight fast (≥ 10 h) for baseline assessments of body composition, resting energy expenditure (REE) and VO_2 peak_ (Fig. 1). Body composition was measured using whole-body fan beam dual-energy X-ray absorptiometry (DEXA) (Hologic QDR Series, Discovery A, Bedford, MA, USA). Following removal of jewellery and metal objects, participants were scanned (~ 180 s) in a supine position. Each scan was automatically analysed with the QDR software; however, the operator manually corrected the trunk and limb regions and an area identifying visceral adipose tissue (VAT). Percentage body fat (%), total body fat (kg) and FFM (kg) are presented as subtotal values excluding head measurements to reduce measurement error.

Following the DEXA scan, participants’ REE was measured using a Moxus Modular Metabolic system (AEI Technologies, IL, USA). Participants were required to lay in a supine position in a dark room for 10 min before the Moxus ventilation hood was placed over their head and shoulders for a further 20 min. Volume flow rate was maintained above 25 L.min^−1^. The first 5 min of data was discarded according to best practice methods [37]. REE was calculated towards the end of the 20 min period, averaged over a 5 min stable period where coefficient of variation for VO_2_ and VCO_2_ was ≤ 10%. REE (kcal/min) was calculated using the Weir equation where ${\text{REE }}\left( {{\text{kcal}}/{\min}} \right)\, = \,\left( {3.94 \times {\text{VO}}_{2} \left( {{\text{L}}.{\min}^{{ - {1}}} } \right)} \right)\, + \,\left( {{1}.{11}\, \times \,{\text{VCO}}_{{2}} \left( {{\text{L}}.{\min}^{{ - {1}}} } \right)} \right)$ [38]. This number was then used to calculate REE (Kcal/day) over an entire day.

Participants also performed a progressive exercise test to exhaustion on an electronically-braked cycle ergometer (Lode Corival, Lode B.V, Groningen, Holland) to determine VO_2 peak_ using the Moxus Modular Metabolic system (AEI Technologies, IL, USA). The test consisted of cycling initially at 60 W, followed by sequential increments of 35 W every 3 min until cadence was reduced to < 50 rpm, at which point the test was terminated. VO_2 peak_ (L.min^−1^) was taken as the highest value obtained in the last 30 s of the test. This value was then expressed relative to fat-free mass (FFM) (kg) obtained from the DEXA scan to give a VO_2 peak_ (ml.min^−1^. kg (FFM)^−1^) normalised for fat-free mass body weight allowing males and females to be compared directly.

Following pre-experimental testing, participants were provided with a 3-axis activity monitor (wGT3X-BT, ActiGraph, Pensacola, FL, USA) and a food diary to record habitual activity levels and food intake, respectively. Participants were advised to continue habitual activity levels whilst wearing the activity monitor on their right waist for a duration of 3 days (2 weekdays and 1 weekend day). Participants were provided with detailed written and verbal instructions on how to complete the 3-day food diary (2 weekdays and 1 weekend day), including information on quantity, food preparation, brand information and cooking methods. Habitual diets were later analysed manually for total daily intake (kcal) and macronutrient breakdown (Table 2).

**Table 2:** Estimated energy requirements and actual HFHC energy intakes

	Males (*n* = 11)	Females (*n* = 10)
Self-reported habitual intake (kcal)	2639 ± 453	2193 ± 407
Self-reported diet fat intake (%)	33 ± 2	34 ± 7
Estimated TEE (kcal)	2653 ± 407	2259 ± 349
Required HFHC intake (kcal)	3979 ± 610	3389 ± 523
Actual HFHC intake (kcal)	4063 ± 669	3464 ± 357
HFHC diet fat intake (%)	65 ± 1	65 ± 1
HFHC diet saturated fat intake (% of overall fat)	37 ± 3	37 ± 2

Data provided as mean ± SD

*TEE* total energy expenditure

### Experimental design

Participants were advised to consume their habitual diet 7 days prior to pre-testing. Experimental testing was conducted pre- and post-7-day HFHC diet (Fig. 1). Pre-testing for female participants occurred between days 1–6 of the menstrual cycle (follicular phase) to minimise the impact of fluctuations of sex hormones on the results. Participants attended the laboratory following an overnight fast (≥ 10 h) after abstaining from caffeine, alcohol and vigorous exercise the day before testing. The following measurements were conducted pre- and post-HFHC diet intervention in addition to a DEXA scan, as previously described.

#### Arterial stiffness

Participants rested in a supine position for 15 min before 3 brachial artery blood pressure measurements were conducted using an automated sphygmomanometer (GE CARESCAPE V100-1 Patient monitor, GE Healthcare, Chicago, USA). Arterial stiffness was measured using central (carotid– femoral) pulse wave velocity (aPWV) using a semi-automated device and software (SphygmoCor, AtCor Medical, Sydney, Australia) as previously described [39]. Systemic wave reflection was investigated using pulse wave analysis to produce an augmentation index with the semi-automated device and software (SphymoCor; [39]). The augmentation index was normalised to a heart rate of 75 bpm (AI_x_@75 bpm) to account for confounding influence of heart rate. All measurements were made in triplicate.

#### Oral glucose tolerance test

A 20G cannula (BD Venflon™, BD, Oxford, England) was inserted into an antecubital vein to allow for repeated blood sampling during the oral glucose tolerance test (OGTT). A 10 ml blood sample was obtained at baseline before participants consumed a 75 g glucose drink made with 225 ml of water. Subsequent blood samples (10 ml) were obtained at 15, 30, 45, 60, 90 and 120 min following glucose ingestion. Blood samples were divided equally between vacutainers containing EDTA or a clotting agent (silicon and micronized silica) (BD, Oxford, England) to allow for the separation of plasma and serum, respectively. Prior to centrifugation, EDTA tubes were stored on ice, whereas serum tubes were left at room temperature until clotting had occurred. Blood tubes were centrifuged at 1000 g for 10 min at 4 °C before being stored at – 80 °C for subsequent analysis.

#### Metabolic flexibility

Prior to the OGTT, participants rested in a supine position for 20 min in a dark room with a ventilation hood placed over their head and shoulders (Moxus Modular Metabolic System, AEI Technologies, IL, USA). VO_2_ and VCO_2_ were measured from the collected expired air and used to calculate the respiratory exchange ratio (RER). The first 5 min of data was discarded according to best practice methods [37]. Fasting RER was averaged over a 5 min stable period where coefficient of variation for VO_2_ and VCO_2_ was ≤ 10%. The Moxus ventilation hood was removed for a brief period (≤ 2 min) to allow participants to consume the glucose beverage before being returned over their head and shoulders for the remainder of the OGTT. The Moxus volume flow rate was maintained at > 25 L.min^−1^. For the remaining OGTT, RER was averaged over 5 min intervals. Peak RER following glucose ingestion was identified as the maximum RER obtained at any 5 min interval during the 120 min OGTT. The time that peak RER was obtained was recorded and used in the following equation to calculate metabolic flexibility.

$${\text{Metabolic flexibility}} = \frac{{{\text{Peak RER}} - {\text{Fasting RER}}}}{{\text{Time to reach peak RER}}}$$

#### Blood analyses

Serum samples were analysed for glucose (all time points during OGTT) and fasting non-esterified fatty acids (NEFA), triglycerides, cholesterol, aspartate aminotransferase (AST), alanine aminotransferase (ALT) and gamma-glutamyltransferase (GGT) concentrations using commercially available spectrophotometric assays (Randox) with a semi-automatic analyser (Randox Daytona RX, Randox, Crumlin, UK). Plasma samples from the OGTT were analysed for insulin concentrations with a commercially available direct insulin ELISA kit (Fisher Scientific, Loughborough, UK).

#### Calculations

Glucose area under the curve (AUC) and insulin AUC during the OGTT were calculated using the trapezoidal rule with zero as baseline.

Whole-body insulin sensitivity was calculated using the Matsuda insulin sensitivity index (ISI) [40] with the following equation;

$${\text{Matsuda ISI}} = \frac{10,000}{{\sqrt {\left( {{\text{FPG }} \times {\text{FPI}}} \right) \times \left( {{\text{MPG}} \times {\text{MPI}}} \right)} }}$$

Hepatic and muscle insulin resistance indexes were estimated using the following equations from Abdul-Ghani et al. [41];

$${\text{Hepatic insulin resistance index }} = {\text{ PG AUC}}_{{0{-}30{\min}}} \times {\text{PI AUC}}_{{0{-}30{\min}}}$$

$${\text{Muscle insulin resistance index } = \text{ }}{{{\text{dG}}} \mathord{\left/ {\vphantom {{{\text{dG}}} {{\text{dt}}}}} \right. \kern-\nulldelimiterspace} {{\text{dt}}}} \div {\text{MPI}}$$

where FGP is fasting plasma glucose, FPI is fasting plasma insulin, MPG is mean plasma glucose during the OGTT, MPI is mean plasma insulin during the OGTT, PG AUC_0–30 min_ is plasma glucose area under the curve from 0 to 30 min, PI AUC_0–30 min_ is plasma insulin area under the curve from 0 to 30 min and dG/dt is rate of change in plasma glucose from its peak to its nadir.

### Seven day HFHC diet

Although habitual dietary intake was assessed (Table 2), there are known issues with participants under-reporting food intake [42] and therefore the energy value of the HFHC was determined based on REE and physical activity levels. The participants were highly active, as measured objectively by accelerometry and subjectively by the International Physical Activity Questionnaire (IPAQ) [43]. As fitness and activity levels were similar between sexes, the same activity factor of 1.7 was multiplied by REE for both males and females to calculate total energy expenditure (TEE), before being multiplied by 1.5 to calculate each individual’s energy intake required during the HFHC diet (Table 2). The diets were designed so that fat contributed 65% of total energy intake, with approximately 37% of fat intake derived from saturated fat. The remaining energy was provided from carbohydrate (~ 17%) and protein (~ 18%). All food was purchased and provided by the research team, in addition to a set of scales provided to the participants so all food items could be weighed correctly. Food for the diet was selected such that it would require minimal preparation and cooking by each participant. Where cooking was required, participants were given strict instructions on how to cook the food and were provided with the quantity of cooking oil/butter where necessary.

To increase compliance to the diet, all participants were provided with a HFHC diet plan prior to the intervention to ensure palatability with all foods. Participants were instructed of the importance to consume all food on their HFHC dietary plan and to not consume any other food products. Participants were advised to report and return any food items that they could not consume during the diet so that energy intake and macronutrient percentage could be adjusted as necessary. All participants were required to record the food items and note the time they consumed them in an individual food diary. Each participant was instructed to continue their habitual physical activity (PA) levels and wore the 3-axis activity monitor (wGT3X-BT, ActiGraph, Pensacola, FL, USA) to ensure habitual PA levels remained constant.

### Statistics

An independent *t* test was used to confirm equal matching of male and female participants for age, BMI and VO_2 peak_ [ml.min^−1^.kg (FFM)^−1^]. All metabolic and cardiovascular functional outcomes were analysed using a two-factor repeated measures ANOVA, with the between-subjects-factor ‘sex’ (males vs. females) and within-subjects-factor ‘HFHC diet’ (pre- vs. post-diet). Significant main effects and interactions were assessed post hoc using Bonferroni adjustment analysis.

## Results

### Subject characteristics

At baseline, there was no significant difference between males and females for age, BMI and VO_2peak_ (ml.min^−1^.kg (FFM)^−1^) (*P* > 0.05; Table 1). Of the females who completed the HFHC diet, 4 were using the combined pill contraceptive method.

### Energy intake and macronutrient composition during the HFHC diet

All participants returned their HFHC diet plan with confirmation of times that they had consumed the food throughout the 7 days. All participants adhered to the HFHC diet and any food that was not consumed by the participants was notified to the researchers and returned to the laboratory to allow HFHC diet calculations to be adjusted. Both sexes consumed an equal proportion of dietary fat as saturated fat (Table 2). All participants continued their habitual exercise regime throughout the 7-day HFHC diet period. Average daily physical activity energy expenditure calculated from the 3-axis activity monitor (wGT3X-BT, ActiGraph, Pensacola, FL, USA) revealed no significant differences between habitual physical activity levels (535 ± 224 kcal) and during the HFHC (510 ± 229 kcal) for either sex (*P* = 0.552).

### Body composition

Prior to the HFHC diet (baseline), males had significantly higher body mass than females (*P* < 0.009), but there was no effect of the HFHC diet on body mass for either sex (*P* = 0.121). Although matched for BMI, at baseline, females had a significantly higher body fat percentage (*P* < 0.001) and total fat mass (*P* = 0.003; Table 3). Conversely at baseline, males had significantly higher FFM (*P* < 0.001), visceral fat (*P* < 0.001) and a higher trunk to leg fat mass ratio (*P* < 0.001), whereas trunk fat mass was not different between sexes (*P* = 0.101; Tables 2 and 3). Following the HFHC diet, there was a trend towards an increase in total fat mass (*P* = 0.056) which was similar for males (+ 1.8%) and females (+ 2.3%; Table 3). Following the HFHC diet, there was a significant increase in trunk to leg fat mass ratio in males only (+ 5.1%; HFHC diet x sex interaction, *P* = 0.039, Table 3). After the HFHC, there were no alterations in FFM, body fat percentage, visceral fat or trunk fat mass in either sex.

**Table 3:** Effect of 7-days HFHC diet on body composition

	Males		Females	
	Pre (*n* = 11)	Post (*n* = 11)	Pre (*n* = 10)	Post (*n* = 10)
Body mass (kg)	72.9 ± 4.9*	73.2 ± 5.2	63.7 ± 9.3	63.9 ± 2.9
BMI (kg. m^−2^)	23.2 ± 1.4	23.3 ± 1.4	23.2 ± 1.5	22.8 ± 2.4
FM (kg)	11.7 ± 3.1*	11.9 ± 3.0	17.3 ± 4.1	17.7 ± 4.0
FFM (kg)	58.6 ± 5.5*	57.9 ± 1.2	43.2 ± 5.9	40.3 ± 10.0
Body fat (%)	16.8 ± 4.0*	16.9 ± 3.8	28.4 ± 3.6	29 ± 3.3
Trunk fat mass (kg)	5.4 ± 1.4	5.6 ± 1.4	6.7 ± 2.0	6.6 ± 2.0
Trunk/leg fat ratio	0.87 ± 0.13*	0.92 ± 0.17^‡^	0.63 ± 0.09	0.63 ± 0.09
VAT (g)	308 ± 45*	295 ± 47	174 ± 71	177 ± 22

Data are provided as mean ± SD

*BMI* body mass index, *FM* fat mass, *FFM* fat-free mass, *VAT* visceral adipose tissue

*Significant difference vs. females at baseline *P* < 0.05

^‡^Significant interaction effect *P* < 0.05

### OGTT

**Fig. 2:**
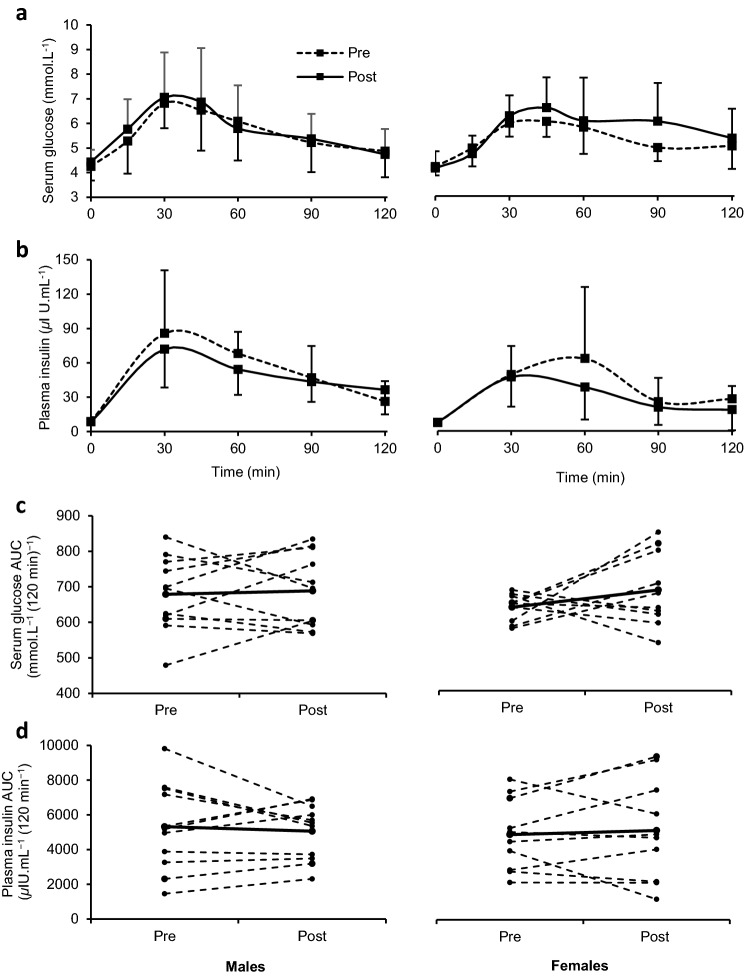
Effect of 7-days HFHC diet on glucose and insulin values during an OGTT in males and females. No sex differences or effects of the HFHC diet on serum glucose levels (**a**) or plasma insulin values (**b**) during the OGTT. The AUC during the OGTT shows no sex differences or effects of the HFHC diet for serum glucose (**c**) or plasma insulin (**d**). For AUC graphs (**c** and **d**) solid black line represents mean value whereas dashed lines show individual responses

At baseline, fasting plasma glucose concentrations were similar between males (4.3 ± 0.6 mmol.L^−1^) and females (4.2 ± 0.4 mmol.L^−1^ L; *P* = 0.766) and after the HFHC diet, fasting glucose did not change in either sex. There were no differences in fasting serum insulin concentrations between males (7.1 ± 3.9 μlU.mL^−1^) and females (7.5 ± 4.0 μlU.mL^−1^; *P* > 0.05) and this did not change following the HFHC diet in either sex. Plasma glucose AUC and insulin AUC during the OGTT were not significantly different between sexes (*P* > 0.05) and not altered following the HFHC diet in either sex (*P* > 0.05; Fig. 2).

#### Insulin sensitivity

**Fig. 3:**
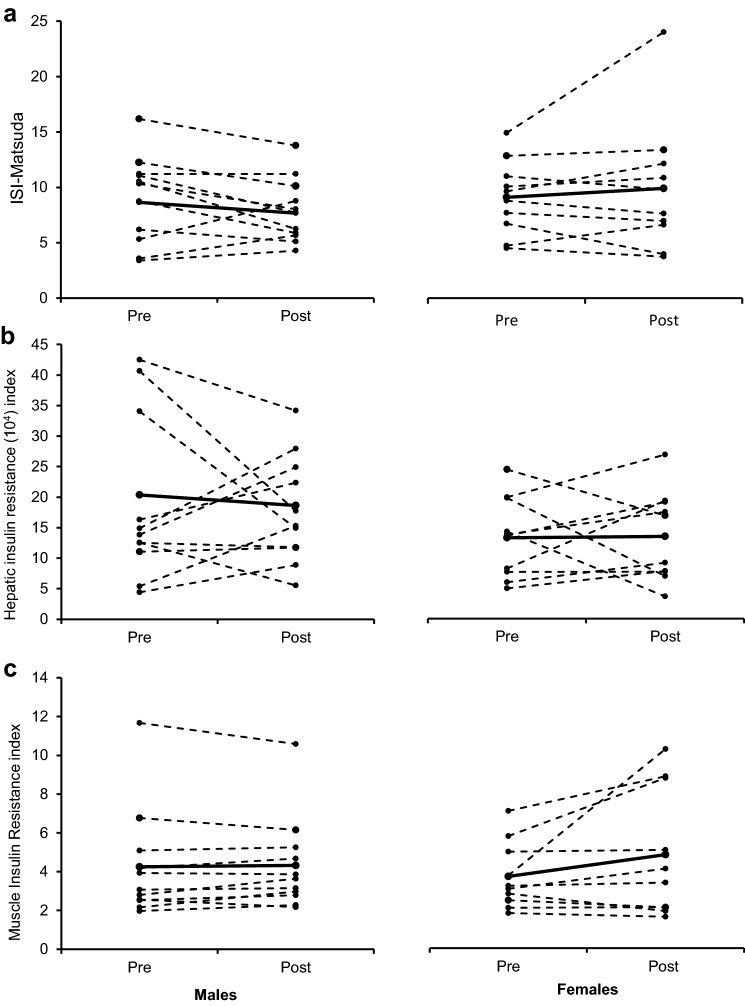
Effect of 7-days HFHC diet on indices of insulin sensitivity measured with OGTT. No sex differences or effects of HFHC diet were seen on the Matsuda index (**a**), hepatic insulin resistance index (**b**) and muscle insulin resistance index (**c**) in males and females. Solid black line represents mean value whereas dashed lines show individual responses. Matsuda index was derived from Matsuda and DeFronzo [40] whilst hepatic and muscle insulin resistance indexes were derived from Abdul-Ghani et al. [41]

In an attempt to decipher changes in insulin sensitivity dependent on origin (liver or muscle), glucose and insulin values from the OGTT were also used to predict hepatic and muscle insulin resistance using the calculations proposed by Abdul-Ghani et al. [41], in addition to the Matsuda ISI (46), which is a measure of whole-body insulin sensitivity. At baseline, there were no significant differences between sexes for the Matsuda ISI (*P* = 0.844), hepatic insulin resistance (*P* = 0.211) or muscle insulin resistance (*P* = 0.584; Fig. 3). After the HFHC diet, there were no significant changes in any of the aforementioned measures of insulin sensitivity in males nor females (Fig. 3).

#### Metabolic flexibility

At baseline, there were no significant differences in fasting RER between males (0.81 ± 0.07) and females (0.76 ± 0.08; *P* = 0.189). Following the HFHC diet, there was a trend towards a reduction in fasting RER, which was reduced similarly between males and females post-HFHC diet (0.78 ± 0.08 and 0.74 ± 0.07 for males and females, respectively; *P* = 0.052). At baseline, peak RER during OGTT was similar between males (0.94 ± 0.07) and females (0.91 ± 0.16; *P* = 0.629). Following the HFHC diet, there was a trend towards a reduction in peak RER for males (0.91 ± 0.07) and females (0.86 ± 0.08; *P* = 0.070). The time it took to reach peak RER during the OGTT was similar between males (88 ± 6 min) and females (90 ± 7 min; *P* = 0.644) and this did not change after the HFHC diet.

**Fig. 4:**
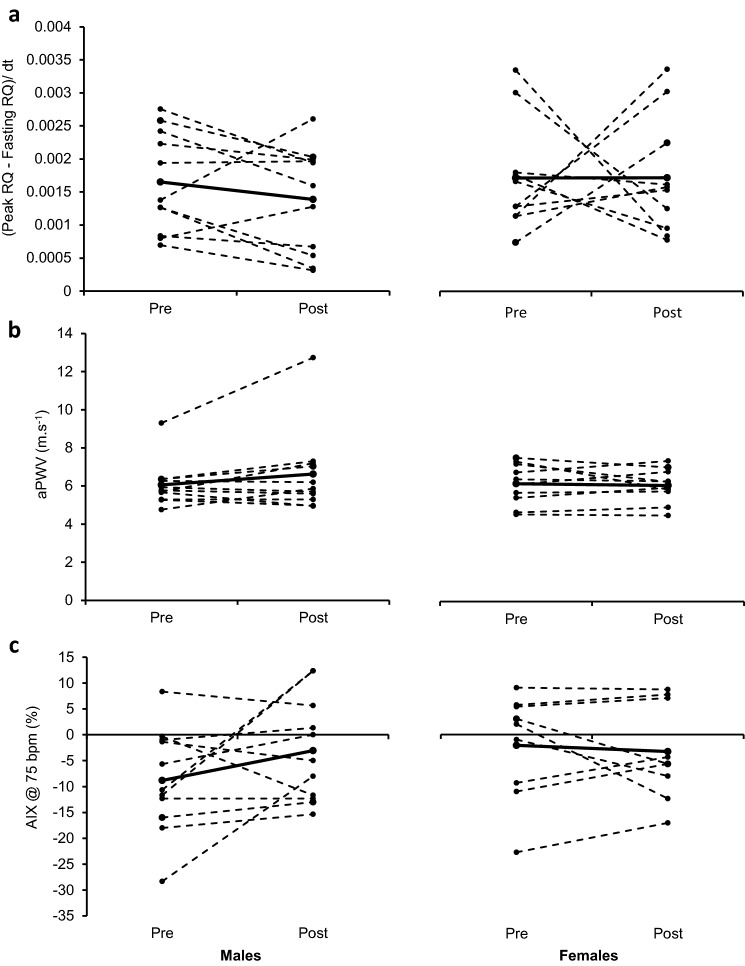
Effect of 7-days HFHC diet on metabolic and cardiovascular functional outcomes. No sex differences or effects of HFHC diet were observed on metabolic flexibility (Peak RQ−Fasting RQ)/dt) (**a**), arterial stiffness (aPWV) (**b**) and augmentation index (@ 75 bpm) (**c**) in males and females. Solid black line represents mean value whereas dashed lines show individual responses

Metabolic flexibility was determined as the rate of change from fasting RER to peak RER during the OGTT. At baseline, there was no significant difference between males and females for metabolic flexibility (*P* = 0.877) and this was unaltered following the HFHC diet in both sexes (*P* > 0.05; Fig. 4a).

#### Arterial stiffness

AI_x_@75 bpm was not significantly different at baseline between sexes (*P* = 0.162) and was unaltered following the HFHC diet (*P* = 0.318; Fig. 3b). aPWV was also not significantly different at baseline between sexes (*P* = 0.826) and was unaltered after the HFHC diet (*P* = 0.307; Fig. 4c).

#### Serum lipids and liver enzymes

At baseline, there were no significant differences between sexes for serum NEFA (*P* = 0.912), cholesterol (*P* = 0.878) and triglycerides (*P* = 0.315; Table 4). After the HFHC diet, there was a significant increase in cholesterol (*P* = 0.049) which was similar between males (+ 8.1%) and females (+ 6.3%; Table 4). Triglyceride and NEFA concentrations remained unchanged following the HFHC diet for both sexes (*P* = 0.685; Table 4).

**Table 4:** Effect of 7-days HFHC diet on serum lipids and liver enzymes

	Males		Females	
	Pre (*n* = 11)	Post (*n* = 11)	Pre (*n* = 10)	Post (*n* = 10)
Serum Lipid concentrations				
NEFA (mmol.L^−1^)	1.16 ± 0.43	1.05 ± 0.25	1.17 ± 0.26	1.37 ± 0.46
Cholesterol (mmol.L^−1^)	3.56 ± 0.86	3.85 ± 0.63^†^	3.95 ± 0.59	4.20 ± 0.58†
Triglycerides (mmol.L^−1^)	0.81 ± 0.92	0.70 ± 0.45	0.51 ± 0.17	0.59 ± 0.25
Liver enzymes				
AST (U.L ^−1^)	35.82 ± 7.86	34.64 ± 13.66	28.10 ± 4.52	28.25 ± 17.77
ALT (U.L^−1^)	35.36 ± 19.80*	35.05 ± 16.77	16.15 ± 7.35	16.80 ± 7.30
AST/ALT ratio	1.17 ± 0.43*	1.08 ± 0.39	1.75 ± 0.47	1.62 ± 0.36
GGT (U.L^−1^)	21.65 ± 11.87	21.13 ± 8.41	15.28 ± 12.15	11.56 ± 5.39

Data are provided as mean ± SD

*ALT* alanine aminotransferase, *AST* aspartate aminotransferase, *GGT* Gamma-glutamyltransferase

*Significant difference vs. females at baseline *P* < 0.05

^†^Significant main effect for the HFHC diet *P* < 0.05

Circulating liver enzymes AST, ALT and GGT were used as markers of liver damage. At baseline, there was no significant difference in AST concentration between sexes (*P* = 0.152); however, males had significantly higher ALT concentrations (*P* = 0.006) which resulted in lower AST/ALT ratio compared to females (*P* = 0.009). There was also a trend towards higher GGT concentrations in males compared to females (*P* = 0.059; Table 4). After the HFHC diet, there were no significant changes in levels of AST, ALT, AST/ALT ratio or GGT in either sex (*P* > 0.05; Table 4).

## Discussion

This is the first study in humans to investigate sex differences in measurements of metabolic and cardiovascular health indicators in response to a 7-day HFHC diet. The results show there was no effect of the HFHC diet on metabolic and cardiovascular outcome measures in either sex. This highlights that young, physically active humans of both sexes who have good levels of cardio-respiratory fitness can tolerate a 7-day HFHC diet without measurable impairments in metabolic or cardiovascular health.

Of the metabolic and cardiovascular functional outcomes measured, the only variables that changed in both sexes were body fat and total cholesterol. We are the first to study and quantify changes in fat mass following a short-term (5–7 day) HFHC diet and determine sex-specific changes in a well-matched cohort. The results suggested a trend towards an increase in body fat following the HFHC diet which occurred in both sexes. In comparison to total adiposity, abdominal adiposity and in particular elevated visceral fat, are more closely associated with insulin resistance and T2D [22, 44]. In line with previous findings [45–47], at baseline visceral fat stores were higher in males compared to females, but this did not change following the HFHC diet. Trunk fat measured by the DEXA scan represents overall abdominal adiposity rather than specific visceral fat. There were no differences between males and females at baseline for trunk fat and following the HFHC diet trunk fat was not changed, but there was an increase in trunk to leg fat mass ratio which increased only in males (Table 3). This change in unfavourable fat accumulation ratio occurred after only 7 days of a HFHC diet but without any decrements in insulin sensitivity. It is likely that more chronic positive energy balance would exacerbate this fat accumulation in males and start to contribute to the development of metabolic disorders.

There was a trend towards a decrease in fasting RER following the HFHC diet with the difference between the sexes not being apparent. There was a large variability between participants and given the large reduction observed in RER, significance would have likely been observed if a larger cohort had been studied. Obese insulin-resistant individuals have previously been shown to exhibit lower metabolic flexibility in comparison to lean insulin-sensitive individuals [13]. In our study, metabolic flexibility was measured as the rate of change from fasting RER to peak RER during the OGTT. As there were no measurable changes from baseline following the 7-day HFHC diet in either sex, this indicates that both the females and the males were able to maintain metabolic flexibility. The current study is the only work to investigate and show no sex differences in metabolic flexibility following a HFHC diet in young healthy active individuals. Whilst metabolic inflexibility is a characteristic of insulin-resistant states, our data suggest metabolic flexibility is unlikely to be compromised following an acute insult of high-fat caloric excess in individuals with high cardio-respiratory fitness.

Overall, 7 days of HFHC diet did not induce changes in whole-body insulin sensitivity (Matsuda ISI), or indices of hepatic or muscle insulin resistance. This was somewhat surprising considering previous studies have reported a reduction in measurements of insulin sensitivity following the same 7-day HFHC diet in young, healthy lean individuals [35, 36, 48, 49]. The current study was novel, however, in measuring and characterising high cardio-respiratory fitness and high physical activity levels of the participants. Longitudinal studies have shown that high cardio-respiratory fitness can reduce the risk of T2D [50] and all-cause mortality [51]. Furthermore, it has been shown that high cardiorespiratory fitness reduces the risk of all-cause mortality in males who have a high BMI in comparison to males who have a normal BMI but have low cardio-respiratory fitness [52]. Low cardio-respiratory fitness is, therefore, established as an independent risk factor for all-cause mortality and may be a stronger risk factor than increased adiposity. We propose the high cardio-respiratory fitness the current cohort possessed may have protected them from the negative effects imposed from the 7-day HFHC diet in this study. To investigate this, future research should compare the metabolic and cardiovascular responses to a HFHC diet in young healthy lean participants with either low or high cardio-respiratory fitness. This study was also unique in considering the physiological variable of sex on the effects a HFHC diet by matching male and female cohorts for age, BMI and cardiorespiratory fitness. We show for the first time there were no sex differences in their response to a HFHC diet when young, healthy participants are investigated.

This was the first study to measure arterial stiffness response to a HFHC diet. Here, it was demonstrated that similar to insulin sensitivity, a 7-day HFHC diet does not appear to be of sufficient duration to impair this cardiovascular functional outcome measure in either sex of young healthy participants. There were also no increases in the circulating concentrations of liver enzymes, AST, ALT and GGT which are early biomarkers of lipid accumulation in the liver [53]. It should be noted that males had a higher ALT concentration resulting in a lower AST/ALT ratio at baseline and after the HFHC diet. Elevated ALT is associated with fatty liver and non-alcoholic fatty liver disease but does not define a specific disease [54]. The reported upper limit of normal of ALT varies greatly between laboratories and sexes (35–79 U.L^−1^ for males and 31–55 U.L^−1^ for females) [55], and although it is established that healthy males have higher ALT concentrations compared to healthy females [56], the mechanisms underpinning this differences are unknown [57]. The two-fold greater ALT concentration in males compared to females in the present study reflects the available epidemiological data [54–57] but is not elevated to an extent which might suggest signs of NAFLD. We anticipated to see increases in the plasma concentration of both triacylglycerol and fatty acids as markers of lipid overload; however, none of these plasma lipid concentrations increased following the HFHC diet in either sex (Table 4). We did, however, observe a significant increase in the plasma total cholesterol concentration in both sexes following the 7-day HFHC diet (Table 4), contradicting previous findings showing no change in total cholesterol following 5-day HFHC diet in males only [33]. Unfortunately, we were unable to interrogate this further to understand whether these increases were due to changes in HDL and/or LDL cholesterol.

Obesity is often associated with reduced PA levels and consumption of HFHC diets. A recent systematic review concluded that HFHC diet studies can often influence participant’s PA levels, which may impact changes in weight gain [58]. In the current study, all participants exhibited high habitual PA levels with the IPAQ and this was largely due to participants participating in regular structured exercise. Participants continued their regular exercise regime throughout the 7-day HFHC diet period and habitual daily energy expenditure measured with accelerometry was the same before and during the diet period. This, in addition to good levels of cardio-respiratory fitness could explain why both male and female participants in our study were able to compensate for the potential negative effects of the 7-day HFHC diet. Another study showed that the combination of both reduced PA and HFHC diet led to larger (significant) reductions in insulin sensitivity in healthy young males [59]. A limitation of the present study is that only young participants with good levels of cardio-respiratory fitness were recruited. Further work is now required to determine whether good levels of cardio-respiratory fitness in both young and older adults compensates the effects of a HFHC.

In summary, this study observed no main effects of a 7-day HFHC diet on metabolic and cardiovascular functional outcomes in lean young healthy individuals of either sex with high levels of cardio-respiratory fitness. More research needs to be conducted to examine if the high cardio-respiratory levels protected both sexes from the expected negative effects of the HFHC diet. Despite participants maintaining high levels of PA, the male cohort did experience an increase in trunk to leg fat mass ratio following the HFHC diet, which may be exacerbated with reduced PA. Although epidemiological studies have shown that there is a reduced prevalence of insulin resistance and T2D in females, the underlying mechanisms of this sex difference are currently understudied and not understood. We conclude that future research should investigate whether a repeat of the current study in males and females with a lower cardio-respiratory fitness will exacerbate the negative metabolic and cardiovascular functional outcomes and thus reveal the mechanisms leading to a higher T2D risk in males than in females.
